# Regional Anaesthesia Approaches in Head and Neck Surgery: Current Evidence and Clinical Applications

**DOI:** 10.3390/jcm15103569

**Published:** 2026-05-07

**Authors:** Antonino Maniaci, Mario Lentini, Maria Stella Di Modica, Federica Maria Parisi, Carlos Chiesa-Estomba, Jerome Rene Lechien, Giuseppe A. G. Lombardo, Matthew White, Luigi La Via

**Affiliations:** 1 Department of Medicine and Surgery, University of Enna “Kore”, 94100 Enna, Italy; mario.lentini@unikore.it (M.L.); mariastella.dimodica@unikorestudent.it (M.S.D.M.); federicamaria.parisi@unikore.it (F.M.P.); giuseppe.lombardo@unikore.it (G.A.G.L.); 2 Asp 7 Ragusa, Ospedale Maggiore Modica, 97015 Ragusa, Italy; 3 Department of Otorhinolaryngology-Head and Neck Surgery, Hospital Universitario Donostia, 20001 San Sebastian, Spain; chiesaestomba86@gmail.com; 4 Department of Surgery, UMONS Research Institute for Health Sciences and Technology, University of Mons, 7000 Mons, Belgium; jerome.lechien@umons.ac.be; 5 Plastic Surgery and Burn Unit, “Cannizzaro” Hospital, 95100 Catania, Italy; 6 Division of Otolaryngology, Faculty of Health Sciences, University of Cape Town, Capetown 6665, South Africa; matthewwhite86@yahoo.com; 7 Department of Anaesthesia and Intensive Care, University Hospital Policlinico “G. Rodolico-San Marco”, 95100 Catania, Italy; luigilavia7@gmail.com

**Keywords:** regional anaesthesia, head and neck surgery, cervical plexus block, ultrasound-guided, opioid-sparing, analgesia, ERAS

## Abstract

General approaches of head and neck surgery involve varied procedures with developing perioperative care and a renewed effort on regional anaesthesia (RA) for intraoperative and postoperative analgesia. Due to the rich innervation and focus on enhanced recovery after surgery (ERAS), RA techniques, particularly ultrasound-guided ones, are becoming popular as part of an opioid-sparing multimodal analgesic regimen. However, the evidence base is heterogeneous and synthesised clinical guidance is needed. This narrative review, performed according to the Scale for the Assessment of Narrative Review Articles (SANRA) framework, summarises the existing literature on the role of RA in head and neck surgery, including anatomical basis, types of techniques used for RA, clinical applications, immediate outcomes and implementation. We conducted a comprehensive literature search, including studies published between January 2000 and October 2025 in English, across the PubMed/MEDLINE, Scopus, and Cochrane Library databases. Understanding of the anatomy of cervical plexus (C1–C4) and cranial nerves such as trigeminal V is basic to delineation of techniques into superficial (e.g., SCPB), deep and selective cranial nerve blocks. The evidence about decreases in postoperative pain intensity, opioid consumption (especially 24 h post-op) and decreased length of stay, largely through studies on thyroidectomy, has been consistent for SCPB as an adjunct to general anaesthesia. Ultrasound-guided regional anaesthesia (UGRA) has significantly enhanced precision and safety, reducing risks such as phrenic nerve paresis, although the concern for even higher complication rates remains with deeper or bilateral blocks. Although beneficial outcomes have been demonstrated, the literature is plagued by small and heterogeneous trials, variable block protocols, and a lack of data in complex oncologic resections or reconstructive settings. For successful implementation, there is a need for structured training programmes of anaesthesiologists and surgeons involved in the procedure performing UGRA together, institutional protocols on standardised technique, patient monitoring and outcomes auditing. RA is a useful and safe adjunct to head and neck surgery, providing analgesia in the short term and contributing to improved recovery during the perioperative period. Further studies should be conducted through large-scale, standardised trials to resolve the contributions of blocks in complex surgical cases and implement best practices for both training and clinical integration.

## 1. Introduction

Head and neck surgery is a broad field that includes everything from everyday otolaryngologic procedures to advanced oncologic resections and complex reconstruction. In recent decades, improved surgical technique and minimally invasive/robot-assisted strategies have been matched by refinements in anaesthetic management, resulting in renewed interest in perioperative analgesia and airway safety with a focus on rapid recovery [1]. In this region, anaesthetic care has historically been dominated by general anaesthesia with regional techniques assuming an adjunctive role at best. However, the practice landscape is changing, and regional and selective nerve-block approaches are increasingly being used for intraoperative anaesthesia and postoperative analgesia in head and neck procedures [2].

There are a number of attractive drivers for this transition. First, the head and neck region has rich innervation, and the predictable anatomy of major sensory pathways (e.g., cervical plexus, trigeminal branches, and superficial cervical nerves) offers well-defined targets for blockade [3]. Second, the rising focus on enhanced recovery after surgery (ERAS) and opioid-sparing strategies has motivated the integration of regional anaesthesia into multidisciplinary perioperative pathways. When approached diligently, blocks may help reduce opioid consumption, improve patient-reported pain scores and decrease length of stay [4]. Third, ultrasound-guided regional anaesthesia (UGRA) has matured such that many of the blocks previously regarded as technically challenging or high-risk in the head and neck region are now being performed safely with reproducible success [5].

Despite these favourable trends, several knowledge gaps persist. The evidence base for regional approaches in head and neck surgery is heterogeneous: many reports are small case series, retrospective audits or narrative overviews rather than large randomised controlled trials [2]. In addition, there is ongoing uncertainty about the variability in block techniques; incomplete recording of complications, such as inadvertent vascular injection, phrenic nerve paresis, and airway compromise; and the absence of standardisation around block dosing or timing in relation to surgery.

The underlying hypothesis of this review is that regional anaesthesia, in terms especially of ultrasound-guided cervical and selective nerve blockade techniques, may help to improve perioperative analgesia and reduce opioid requirements in selected head and neck surgical procedures, but these effects are likely to differ depending on the type of block used, complexity of procedure and quality of study.

This narrative review aims to consolidate existing evidence for regional anaesthesia in head and neck surgery according to anatomic/neurophysiological premises, technique classification, clinical applications (outcomes), and implementation strategies.

In particular, we aim to:

(1) Provide a clear and clinically oriented overview of the major regional anaesthetic approaches applicable to head and neck surgery;

(2) Synthesise recent evidence regarding their efficacy and safety, including intraoperative and postoperative analgesic outcomes;

(3) Discuss practical considerations and future directions to support broader integration of these techniques into otolaryngology and head–neck surgery practice.

We focus on use in thyroid and parathyroid surgery, as these are the most prominent indications for regional anaesthesia-supported procedures where evidence is strongest; however, we also discuss emerging or less conducted applications of regional anaesthesia to salivary gland surgery, airway procedures, oncologic resections and reconstructive head and neck surgery, which currently have more limited but pertinent treatment utility.

We seek to educate not only our anaesthesiologist audience but the head–neck surgeon readership as well in a manner that fits with both the editorial philosophy of the *Ear, Nose & Throat Journal* and serves as another successive chapter in writing out a structure for what regional anaesthesia looks like for this subset of surgical cases. Owing to the heterogeneity in techniques and outcome reporting, the review will consider disparities in study design, level of evidence and context (e.g., primary anaesthetic vs. analgesic adjunct). It is designed to fulfil the fundamental tenets of narrative review writing—comprehensiveness, balance, method transparency and clinically useful synthesis. By employing this strategy, we hope to create a digestible but rigorous reference tool that can be used by both clinicians and investigators, making sound decisions about therapy or finding areas for future investigation.

## 2. Materials and Methods

This narrative review was conducted in accordance with the Scale for the Assessment of Narrative Review Articles (SANRA) framework, with the aim of ensuring clarity of rationale, transparency of literature retrieval, balanced presentation of evidence, and critical discussion of study limitations [6]. Because the objective of this manuscript was to provide a clinically oriented synthesis rather than a formal systematic review or meta-analysis, the review process was structured but not designed to meet full PRISMA requirements. Nevertheless, additional methodological details were incorporated in the revised version to improve reproducibility and analytical transparency. In alignment with these principles, this section describes the steps undertaken in the literature retrieval, selection, and synthesis process.

The primary objective of this narrative review was to synthesise the current evidence on regional anaesthesia approaches used in head and neck surgery, with an emphasis on clinical efficacy, safety, and applicability in otolaryngologic and oncologic contexts. Unlike systematic reviews, which aim for exhaustive inclusion and meta-analysis, this review sought to integrate clinical insights, emerging practices, and educational implications to provide a pragmatic overview for both anaesthesiologists and head and neck surgeons.

### 2.1. Literature Search Strategy

A comprehensive literature search was performed across the PubMed/MEDLINE, Scopus, and Cochrane Library databases. The search included studies published in English, reflecting the period of most significant evolution in ultrasound-guided regional anaesthesia (UGRA) and its integration into head and neck procedures.

The search strategy combined controlled vocabulary terms and free-text keywords related to regional anaesthesia and head and neck surgery. Representative search syntax used in PubMed/MEDLINE was as follows:

*(“regional anaesthesia” OR “regional anaesthesia” OR “nerve block” OR “cervical plexus block” OR “superficial cervical plexus block” OR “deep cervical plexus block” OR “intermediate cervical plexus block” OR “superior laryngeal nerve block” OR “recurrent laryngeal nerve block” OR “greater occipital nerve block” OR “sphenopalatine ganglion block” OR “ultrasound-guided regional anaesthesia” OR “ultrasound-guided regional anaesthesia”) AND (“head and neck surgery” OR “otolaryngology” OR “thyroidectomy” OR “parathyroidectomy” OR “parotidectomy” OR “neck dissection” OR “laryngeal surgery” OR “tracheal surgery” OR “carotid endarterectomy” OR “reconstructive surgery”).*

The syntax was adapted as appropriate for Scopus and the Cochrane Library.

Reference lists of key review papers and clinical trials were manually screened to identify additional relevant studies. Duplicates were removed, and only peer-reviewed articles, conference papers with full text, and guideline-based reviews were included.

The final literature search was completed in October 2025.

### 2.2. Inclusion and Exclusion Criteria

Eligible studies were defined as randomised controlled trials, prospective and retrospective cohort studies, case series of a minimum of 10 patients and pertinent papers if they contained review or guideline-based aspects discussing the relevance of regional anaesthesia used in head and neck surgery. Case reports were included only when they provided novel techniques or pertinent safety-related clinical data. Studies limited to dental, ophthalmologic, intracranial neurosurgical, or non-head-and-neck pain indications were excluded. Exclusions were non-English publications, conference abstracts without full text and publications that lacked adequate methodological or clinical details. Case reports were considered only if they described novel or clinically impactful techniques.

These criteria were selected to place greater emphasis on clinically informative literature while avoiding inclusion of anecdotal evidence, except in areas where higher-level evidence was still lacking.

### 2.3. Data Extraction and Synthesis

The following data were extracted for each study included when available: the author and year, study design, clinical setting, sample size, type of regional anaesthesia technique used, whether ultrasound guidance was employed in the same block or procedure as compared to that without ultrasound guidance (where applicable), main analgesic or recovery outcomes measured, complications reported and key conclusions reached. Due to significant heterogeneity in methods, procedure types, and reported endpoints, the results were synthesised narratively rather than pooled quantitatively.

While analysing particular patterns that arose across studies, we focused on the consistency of analgesic benefit (if reported), procedure-specific applicability, safety concerns and notable specific methodological drawbacks that may affect interpretation.

To minimise selection and interpretation bias, studies were independently reviewed by two authors for relevance and methodological rigour. Although SANRA is not a scoring system for primary study quality, its items—such as justification of the article’s importance, balanced presentation of evidence, and appropriate referencing—were applied to ensure scientific credibility and educational value.

### 2.4. Study Selection Process

**Figure 1:**
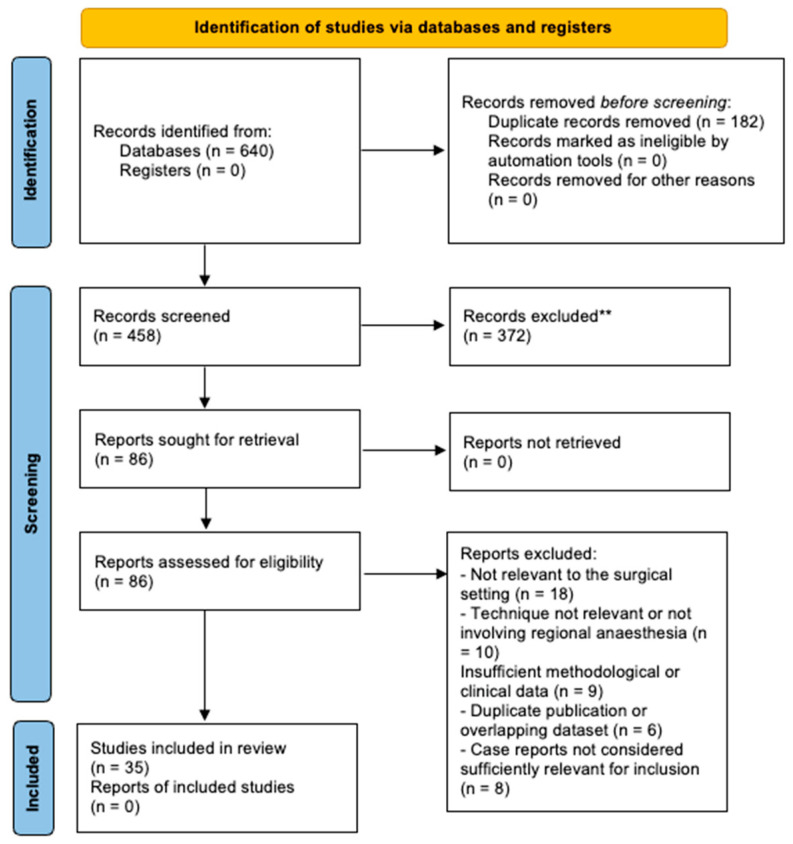
PRISMA flow diagram of study selection. ** records excluded after screening.

Records identified through electronic search were screened in a stepwise manner based on title, abstract and full-text eligibility. Screening: Duplicate records were removed before screening. Two authors reviewed all potentially relevant articles for inclusion independently, with disagreements resolved through discussion and consensus. At the full-text stage, publications deemed irrelevant to head and neck surgery, not pertaining to regional anaesthesia techniques or without sufficient methodological detail were excluded. A flow diagram of the process for identification, screening, eligibility and inclusion represents the entire process (Figure 1).

### 2.5. Qualitative Appraisal of Included Evidence

A formal tool for risk-of-bias was not used due to the highly narrative nature and heterogeneity of the literature included, but a structured qualitative appraisal was performed for the principal studies that made part of this review. Each study was evaluated by design, sample size, surgical subgroup, use of ultrasound guidance, comparators, outcome reporting and significant methodological limitations. Specific sources of potential bias were small sample size, single-centre design, non-randomised allocation, inconsistent block protocols, heterogeneous perioperative analgesic regimens and short follow-up. This review was utilised to inform a more analytical interpretation of the evidence and to delineate stronger findings from those that are in a preliminary phase.

Specifically, in the synthesis, empirical evidence from randomised controlled trials and systematic reviews/meta-analyses received greater interpretive importance; observational studies/case series were included to provide hypothesis-generating or contextual evidence, especially when comparative data are limited in surgical settings.

## 3. Results

### 3.1. Anatomical and Neurophysiological Background

A comprehensive understanding of the anatomical and neurophysiological underpinnings of the head and neck region is foundational for the successful implementation of regional anaesthesia in this surgical domain. Given the complexity and density of neural structures, meticulous attention to the origin, course, innervation, and inter-connections of key nerves is requisite for both efficacy and safety.

#### 3.1.1. Innervation of the Head and Neck: Overview

Sensory and motor innervation to the head and neck region primarily derives from cranial nerves (especially the trigeminal nerve and its branches) and upper cervical spinal nerves via the cervical plexus (C1–C4) [7,8,9]. The trigeminal nerve innervates the facial components, while the cervical plexus and correct spinal nerves provide supplies to anterolateral and posterior cervical distributions, the shoulder girdle, and superficial head structures [10,11,12]. The duality of these systems elucidates not only the potential scope of regional anaesthesia in head and neck surgery but also the complexity of achieving sufficient block coverage.

#### 3.1.2. The Cervical Plexus: Structure and Relevance

The cervical plexus is formed from the anterior rami of C1–C4 spinal nerves, and traditionally consists of superficial (sensory) and deep (motor) components [9]. The superficial branches (great auricular, lesser occipital, transverse cervical and supraclavicular nerves) provide cutaneous sensation to the anterolateral neck, shoulder region and parts of the head [8,13]. The deep branches provide motor innervation to the infrahyoid muscles through the ansa cervicalis and phrenic nerve (C3–C5) for diaphragmatic innervation [14]. Clinically, cervical plexus blocks may be used in medially located thyroid and parathyroid procedures, and carotid and other surgeries of the neck due to the overlap of the superficial sensory constituent with incision sites [7].

Anatomical studies have identified anatomical structures, like the “nerve-point” of the neck (Erb’s point) lying over the mid-posterior margin of the sternocleidomastoid muscle, as an important landmark for accessing the superficial cervical plexus [9,15]. The fascial relationships of the deep cervical fascia also affect local anaesthetic spread and, consequently, block efficacy and risk [11].

#### 3.1.3. Cranial Nerve Contributions

Outside of the cervical plexus, cranial nerves are important. The cranial nerve (V) is split into three branches: ophthalmic (V1), maxillary (V2) and mandibular, which represents V3 generation, after which both trigeminal nerves divide into ophthalmic and maxillary parts [10,11]. For example, V2 innervates the maxilla, hard palate and upper lip; a property that is capitalised on in blocks for maxillofacial surgery [11]. Other involved nerves include the lesser occipital (C2) and greater occipital (C2 dorsal ramus), which innervate posterior scalp areas [11,16,17]. In head and neck surgery, the knowledge of these coinciding sensory fields informs block choice and combination.

#### 3.1.4. Anatomical Variability and Implications for Block Technique

It is important to highlight that great anatomical variations in branching patterns, course of the nerves and communication between plexuses are common. For instance, trapezius muscle studies demonstrated mixed innervation from the cervical plexus and the Spinal Accessory Nerve SAN [18,19], emphasising anatomical complexity. Variability affects block success, anaesthetic distribution, and risk of unintended motor block (e.g., phrenic nerve involvement). Thus, a standardised “one-size-fits-all” approach to neck blocks is inadequate; individualised technique and ultrasound guidance help mitigate such risks.

#### 3.1.5. Neurophysiological Considerations

Regional blocks in the head and neck have to consider both sensory somatic fibres (pain and temperature) and motor fibres when possible from a neurophysiological perspective. The cervical plexus and trigeminal nerve cutaneous branches are primarily somatic, and blockade decreases nociceptive input from surgical incisions. By contrast, blockade of mixed or purely motor nerves—to strap muscles or phrenic nerve, for example—has implications regarding swallowing, airway patency and function of the diaphragm. Real-time assessment of needle-to-nerve relationships under ultrasound-guided visualisation helps to prevent inadvertent spread into unintended structures [18].

#### 3.1.6. Clinical Relevance in Head and Neck Surgery and Integration into Regional Anaesthesia Approach

During head and neck surgical procedures, particularly in the fields of thyroidectomy, parotidectomy, reconstructive flaps and carotid surgery, the superficial sensory branches of the cervical plexus lie within or beneath the surgical incision areas. As such, selective or combined blocks of these nerves can provide effective analgesia and even be a component of the primary anaesthetic plan in selected patients [5]. Such a detailed anatomical understanding facilitates safe mapping out of the block fields, predicting areas of referred pain (for example, cervical plexus to the shoulder), and avoiding complications such as phrenic nerve block or airway compromise. In developing a regional anaesthesia plan for head and neck surgery, the clinician must transpose the proposed surgical field onto its detailed innervation (cutaneous, muscular, and visceral). A superficial cervical plexus block will anaesthetise the skin overlying the lateral neck and a small area of the sternocleidomastoid muscle, but will not track into deeper structures such as the larynx or trachea, which may require additional nerve blocks or infiltration. It is important to know about the cross-innervation from the trigeminal branches to the head and neck region that helps in anticipating analgesic gaps while combining blocks [20,21]. This anatomical landscape acts as a guide in optimising technique and block selection and also serves to explain the failure mechanisms.

#### 3.1.7. Practical Anatomical Landmarks and Take-Home Messages for Clinicians

**Figure 2:**
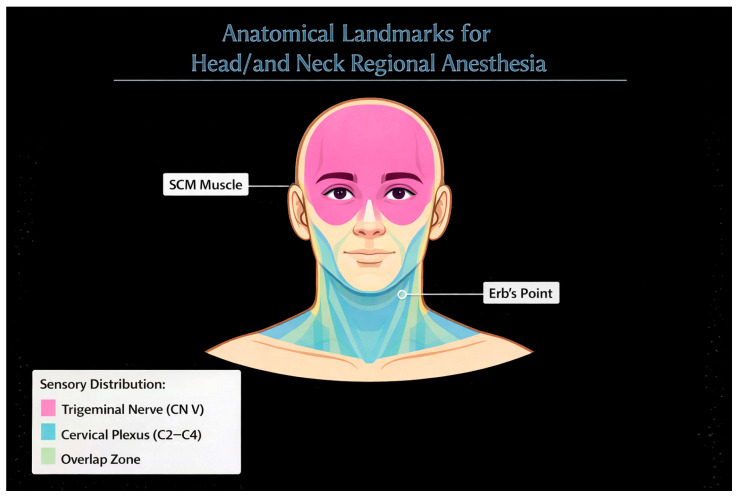
Anatomical landmark for regional blocks.

The performance of regional anaesthesia in the head and neck can only be safely achieved by identifying surface landmarks and deep sonographic landmarks, which rely on the individual block being executed. The main landmark for the superficial cervical plexus block is at the Erb’s point, which is located midway along the posterior border of the sternocleidomastoid muscle (Figure 2).

This is the point at which the more superficial sensory branches of the cervical plexus—the lesser occipital nerve, great auricular nerve, transverse cervical nerve and supraclavicular nerves—leave behind the muscle and are available for blockade. In practical terms, thus, the posterior border of the sternocleidomastoid becomes the crucial surface reference for superficial accesses. For intermediate or deep cervical plexus techniques, the anatomical reference is from the posterior border of sternocleidomastoid to the transverse processes of C2, C3, and C4, at which point they can be identified with respect to their origin. This corresponds to the origin of the cervical nerve root. These layers outline the deeper target area and are very closely related to the prevertebral fascia, being an important space delimiter that influences a local anaesthetic spread. At this level, awareness of the surrounding structures becomes critical as the carotid sheath lies anteromedially, the vertebral vessels run adjacent to transverse foramina and the phrenic nerve courses inferiorly along with the anterior scalene muscle. These relationships explain why more profound injections may offer wider analgesic coverage but are also associated with the increased risk of vascular puncture, epidural spread, or diaphragmatic dysfunction. Other anatomical landmarks come into play, depending on the target of the surgery. The greater cornu of the hyoid bone and thyrohyoid membrane are important landmarks for the superior laryngeal nerve block for procedures to the larynx or supraglottic region. In posterior scalp or retroauricular procedures, the relevant landmark is the course of the greater occipital nerve as it courses along with the superior nuchal line. Particular care should be taken in parotid, auricular or lateral neck cases with regard to the distribution of the great auricular nerve as it courses across the sternocleidomastoid towards the parotid and auricle. Thus, anatomic consideration should be based on a series of topographic references rather than one single landmark: the posterior border of sternocleidomastoid for more superficial sensory access, the cervical transverse processes and fascial planes for deeper plexus techniques and procedure-specific landmarks such as hyoid bone, thyrohyoid membrane or occipital line when/if selective nerve blocks are needed. This difference has clinical implications as each landmark denotes a different depth of injection and a different neural target and risk profile. An accurate definition of these anatomical landmarks enables the clinician to better correlate the block to the surgical field of interest, increase the efficacy of analgesia and decrease the risk of inadvertent spread to important vascular or neural structures.

### 3.2. Overview of Regional Anaesthesia Techniques

In the context of head and neck surgery, regional anaesthesia techniques can be broadly classified into superficial sensory blocks, deep cervical plexus/nerve blocks, and selective cranial or peripheral nerve blocks. The choice of technique is guided by surgical site, desired level of anaesthesia or analgesia, patient comorbidities and the risk–benefit profile. Employing the principles of the SANRA framework—transparency, balanced presentation, and clinical relevance—this section details the classification of regional approaches, contrasts them with general anaesthesia modalities, and traces their evolution from landmark-based to ultrasound-guided methods.

#### 3.2.1. Classification of Techniques

**Figure 3:**
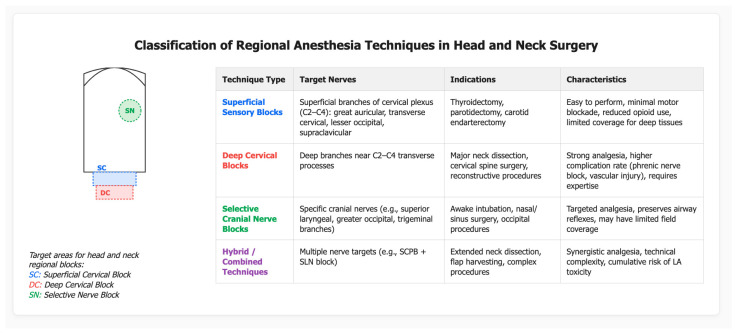
Classification of regional anaesthesia techniques for head and neck surgery based on anatomical target depth, nerve specificity, and clinical applications. SCPB = superficial cervical plexus block; SLN = superior laryngeal nerve; LA = local anaesthetic.

Superficial sensory blocks in the head and neck frequently involve the superficial portion of the cervical plexus (C2–C4) for cutaneous innervation of the anterolateral neck, or selective blocks of nerves such as the greater occipital nerve, the superior laryngeal nerve, or the sphenopalatine ganglion [11,22]. Deep cervical plexus blocks extend to deeper branches supplying neck musculature and may interface with the phrenic or accessory nerves [23] (Figure 3).

Intermediate approaches—sometimes described as between superficial and deep planes—have also been defined [24]. The division underscores the need to tailor the block to the incision depth, anatomical structures involved and the anticipated sensory/motor blockade.

#### 3.2.2. Indications, Contraindications and Comparison with General Anaesthesia

The indications for regional blocks in head and neck surgery include: 

(1) Primary anaesthetic for selected procedures (e.g., superficial neck dissection and thyroid surgery) in appropriate patients; 

(2) Adjunct intraoperative analgesia plus light sedation; 

(3) postoperative analgesic supplementation to reduce opioid use [25].

Blocks may confer benefits such as reduced opioid consumption, earlier mobilisation, and improved recovery profiles [26]. However, contraindications—such as patient refusal, anticoagulation, local infection, and anatomical distortion (from prior surgery or radiotherapy)—must be considered [27]. In comparison with general anaesthesia, regional methods offer potential advantages in high-risk patients, but require operator expertise and vigilant airway management support [28].

#### 3.2.3. Landmark-Based Approaches

In the past, regional head and neck blocks were performed using anatomical landmarks. The classic lateral approach for blocking the cervical plexus was first introduced by Heidenhain and later refined by Winnie and coworkers, which includes palpation of the mastoid process, Chassaignac tubercle (C6 transverse process), and the posterior border of the sternocleidomastoid muscle [29]. Despite being effective, landmark techniques had relatively higher variability and risk of inadvertent vascular punctures or phrenic nerve involvement [30]. Bilateral deep cervical plexus block should be performed without ultrasound, as bilateral phrenic nerve paresis may otherwise result [30]. These concerns prompted the transition toward imaging-guided methods.

#### 3.2.4. Ultrasound-Guided Regional Anaesthesia (UGRA)

The development of ultrasound guidance and its widespread adoption have allowed for the visualisation of neural structures and surrounding vascular anatomy, which can increase the precision and safety of these procedures. Studies show that ultrasound-guided superficial or intermediate cervical plexus blocks are associated with faster onset and fewer complications than landmark techniques [31]. A randomised controlled trial demonstrated that deeper blocks are associated with a greater likelihood of phrenic nerve blockade than superficial ones once depth crosses a critical threshold [28]. UGRA facilitates deposition of local anaesthetic in a targeted manner, hence providing volume sparing benefit with minimal collateral spread and improved block success [32,33]. Notably, in head and neck surgery, this has corresponded to a growing acceptance of blocks for thyroid, parathyroid, carotid and reconstructive operations [11].

#### 3.2.5. Technique Details and Variants

Superficial Cervical Plexus Block (SCPB): Typically performed at the midpoint of the posterior border of the sternocleidomastoid muscle, injecting beneath the superficial fascia. Indications include incisional analgesia for thyroidectomy, lymph-node biopsy, and carotid endarterectomy [11,22].

Intermediate/Deep Cervical Plexus Block (IC/Deep CPB): Marked by deeper injection near C2–C4 transverse processes, sometimes with multiple point injections or catheter placement for continuous analgesia. Wider sensory and minor motor coverage; higher risk of diaphragmatic or brachial plexus spread [23,24] (Table 1).

Selective Cranial/Peripheral Nerve Blocks: Examples include superior and recurrent laryngeal nerve blocks for awake fibre-optic intubation, greater occipital nerve blocks for posterior skull incisions, and sphenopalatine ganglion blocks for nasal and sinus surgery [33].

Combined/Hybrid Techniques: Some authors report combining SCPB with selective nerve blocks (e.g., supraclavicular cutaneous nerves) or infiltration analgesia to achieve multimodal coverage in head and neck oncologic reconstructions [34].

#### 3.2.6. Choosing the Appropriate Technique

There are many factors upon which the choice of block depends: site and depth of surgery (skin vs. deep tissue), expected pain intensity, airway management issues, patients’ comorbidities (especially respiratory function) and logistics for doing the block (time, expertise and equipment) [35,36,37,38,39,40]. The choice of regional technique in head and neck surgery should be based on four practical questions: (1) what is the primary operative territory, (2) what is the perioperative objective, (3) how extensive and deep is the expected nociceptive field, and (4) does the patient have features that increase the risk of cervical plexus block spread or airway-related complications. In practical terms, superficial cervical plexus block is most appropriate for superficial anterior or lateral neck procedures when the main objective is postoperative analgesia and opioid reduction. Intermediate approaches may be considered when somewhat broader soft-tissue coverage is required but a more favourable safety profile than deep injection is desired [35]. Deep or combined techniques should be reserved for carefully selected cases in which broader block density is needed and the patient’s respiratory and anatomical risk profile is acceptable. By contrast, selective airway-related nerve blocks are better matched to awake instrumentation or laryngeal/tracheal procedures, where the primary goal is facilitation of airway management or procedural tolerance rather than broad postoperative analgesia. On the other hand, when a larger flap needs to be harvested or for a deep neck dissection, either a deeper or combined block may be indicated but with increased awareness of possible complications [22,30].

This practical approach to patient stratification would line out three groups. At first, patients undergoing relatively superficial procedures lacking significant pulmonary or anatomical risk factors are appropriate candidates for superficial cervical plexus-derived analgesic techniques as part of routine multimodal care [36]. Second, candidates with a larger expected area of paresthesia above C3–C4 who maintain respiratory reserve can be evaluated for wider regional approaches if the operative field warrants such an approach. Third, patients with contralateral phrenic nerve dysfunction, severe pulmonary disease, prior neck surgery and/or neck irradiation, active local infection or distorted anatomy should be approached cautiously and preferentially have the least invasive technique that adequately covers the relevant field performed or avoid cervical plexus-based techniques altogether when necessary [40,41].

#### 3.2.7. Safety Considerations and Pitfalls

Head and neck regional blocks are associated with distinctive risks because of the sectors that are vascular, neural and airway related in a confined anatomical space. But these risks are not uniform among techniques [31,42]. Available comparative data indicate that superficial and intermediate cervical plexus approaches have improved safety profiles compared to deep or combined techniques, especially with regard to direct needle injury and phrenic nerve involvement. On systematic review of the literature regarding cervical plexus block for carotid endarterectomy, a significantly higher rate of serious block-related complications and a greater rate of conversion to general anaesthesia were found when deep/combined techniques were employed compared with superficial/intermediate approaches [43]. Recent randomised data confirm that depth-dependent diaphragmatic dysfunction is strongest after a deep block [44]. In contrast, intermediate ranges of lower-dose ultrasound-guided may minimise but not remove phrenic involvement [39]. Thus, safety is best viewed along a spectrum of injection depth and injectate spread as opposed to a dichotomous feature of cervical plexus block as a whole.

Regional blocks in the head and neck region bear unique risks: accidental intravascular needle placement (because of the surrounding carotid, vertebral and jugular vessels), local anaesthetic systemic toxicity (due to vascularity), phrenic nerve palsy (specifically with deep plexus block), airway compromise secondary to motor nerve spread, haematoma formation (in patients on anticoagulant therapy) and nerve injury [31,45]. Real-time ultrasound, frequent aspiration, limiting volumes and patient selection all ameliorate many of these risks. Moreover, bilateral deep plexus blocks are not recommended in patients with limited respiratory reserve [29].

The implication that bilateral deep injections are problematic due to the potential for increased phrenic nerve dysfunction, and are particularly worrisome in patients with impaired contralateral phrenics, chronic lung disease, diminished pulmonary reserve and likely postoperative airway compromise should be emphasised [20].

#### 3.2.8. Evidence Summary and Technique Efficacy

Overall, the best evidence supports SCPB as an adjuvant in thyroid surgery, which has been consistently reported in systematic reviews, meta-analyses and several randomised controlled trials to reduce early postoperative pain and consumption of analgesics. The overall magnitude of the benefit, however, is modest and appears limited to the first postoperative 24 h, with considerable between-study heterogeneity in block protocol, comparator regimen, and definition of outcome. In contrast, the data comparing superficial, intermediate, and deep cervical plexus techniques is less homogeneous. Comparative trials suggest that deeper techniques may expand sensory coverage but there is no consistent evidence to support superior analgesia and it can be associated with a less favourable safety profile, including phrenic nerve involvement or inadvertent deep spread. Except for thyroid surgery, most of the literature comes from observational studies, procedural reports, or small case series; thus, generalisability remains uncertain. Thus, the current evidence favours SCPB most conclusively in selected superficial cervical operations but more general claims for use with other techniques or complex surgical indications should be interpreted with caution [35,40]. Comparative trials between superficial vs. deep cervical blocks suggest similar analgesic efficacy, but with lower complication rates for the superficial technique [31].

This trade-off is clinically relevant. Infusion of deeper anatomy has not been accompanied by a jump to a superior overall risk-benefit profile, particularly since superficial or intermediate techniques already propose adequate coverage for many cervical interventions [20,42,43].

More recent trials comparing intermediate cervical plexus vs. erector spinae plane blocks found no superiority in analgesic duration for the cervical technique, albeit quicker performance and onset [26]. Despite these data, many studies remain small, heterogeneous and often observational, underscoring the need for larger standardised trials.

#### 3.2.9. Evolution and Future Directions

Since the introduction of regional anaesthesia techniques into head and neck surgery, blind landmark-based injections have given way to image-guided, precision techniques with improved safety and efficacy. What is the current outlook for regional anaesthesia in 3 to 5 years? Techniques are advancing towards lower volumes, continuous catheters for long-term analgesia and incorporation with enhanced recovery after surgery (ERAS) pathways. Novel techniques such as perineural catheter placement for the flap harvest, fascial plane blocks in the neck region and even robotic or programmed needle guidance for cervical blocks. As ultrasound and clinician training increase, regional anaesthesia is becoming a more important element of the anaesthetic strategy in head and neck surgery.

### 3.3. Evidence-Based Clinical Applications

Regional anaesthesia techniques in head and neck surgery have been considered in a wide variety of procedural contexts, but the volume and quality of the available evidence are highly heterogeneous by surgical subgroup. The highest-quality literature pertains to thyroid and parathyroid surgery, while evidence in salivary gland procedures, airway-related interventions, oncologic resections and reconstructive surgery remains more limited, disparate and frequently observational. For this reason, the following synthesis is structured not only by clinical indication but also based on the relative maturity of evidence existing in each domain.

In the context of clinical decision-making, the literature is most meaningfully evaluated according to perioperative purpose. Regional anaesthesia is mainly used in thyroid and parathyroid surgery to enhance early postoperative analgesia and minimise opioid consumption [46]. In this setting of salivary gland and lateral neck surgery, it could assist analgesia in anatomically accessible superficial fields. How does this differ from the conventional approach? In airway-specific procedures, the primary goal becomes facilitation of awake instrumentation or procedural tolerance rather than routine postoperative pain control. Regional techniques in oncologic and reconstructive surgery are best regarded as parts of multimodal and ERAS-based algorithms rather than stand-alone solutions.

Recently, the clinical utility of regional anaesthesia techniques in head and neck surgery—specifically, the use of superficial cervical plexus block (SCPB) and related approaches—has been increasingly investigated.

#### 3.3.1. Thyroid and Parathyroid Surgery

In head and neck surgery, the most widely studied clinical applications of regional anaesthesia are thyroidectomy and parathyroid surgery, with superficial cervical plexus block (SCPB), unilateral or bilateral, being employed more often as an adjunct to general anaesthesia than as a primary anaesthetic technique [41,42,43,44,45]. Generally, thyroid and parathyroid procedures continue to be the primary procedural indication for cervical plexus-based techniques on the basis of technique alone, although their use is generally limited to superficial anterior neck structures rather than extensive deep dissection.

In this subgroup, the literature is also methodologically stronger than that for other head and neck settings, as it consists of randomised trials and meta-analyses rather than isolated observational reports alone. As such, the strength of conclusions about analgesic benefit is greater for thyroid and parathyroid surgery than for other applications examined here.

From a clinical decision-making standpoint, this subgroup is currently the most directly relevant indication for superficial cervical plexus-based regional anaesthesia as an adjunct to general anaesthesia when early pain reduction and opioid sparing during the onset of surgery are among the perioperative goals.

#### 3.3.2. Parotidectomy, Submandibular and Other Salivary Gland Surgeries

Evidence in parotidectomy, submandibular surgery and other salivary gland procedures is comparatively limited compared with thyroid surgery, but existing data suggest a possible role for cervical plexus and selective peripheral nerve blocks in lateral neck, parotid region and peri-auricular territory operations [47]. While large randomised studies specifically targeting salivary gland surgery are lacking, the anatomical distribution of the superficial cervical branches and auriculotemporal innervation offers a sound basis for selective regional approaches in these operations.

Thus, the available evidence in salivary gland surgery should be considered equal to suggestive rather than conclusive, and the current endorsement for these approaches is based more on anatomical rationale and small clinical studies than high-level comparative evidence.

In addition to anatomical plausibility, parotid surgery is also supported by emerging comparative evidence. A recent prospective randomised study of parotid procedures found that cervical retrolaminar block plus auriculotemporal nerve block provided longer-lasting analgesia and decreased postoperative opioid requirements compared with superficial cervical plexus block plus auriculotemporal block, but with superior performance time and hypotension [48]. There have also been feasibility studies describing sedation and locoregional anaesthesia in parotidectomy, and more recent retrospective work indicates that selected cases of surgery to the parotid gland might be performed using local anaesthesia with conscious sedation in appropriately chosen patients [49]. Taken together, these data suggest that salivary gland surgery is a significant but still more immature surgical application of regional anaesthesia.

#### 3.3.3. Laryngeal, Tracheal and Airway-Related Procedures

Procedures involving the head and neck have particular challenges related to regional anaesthesia given deeper structures, cranial nerve innervation complexities, and airway-related risks. Data available in this area remains sparse but is increasing slowly [33]. In this setting, selective laryngeal nerve blocks are primarily used to facilitate airway instrumentation or targeted airway procedures rather than to provide broad postoperative analgesia. Because of airway-specific safety concerns, these techniques should be considered within a multidisciplinary perioperative strategy.

**Figure 4:**
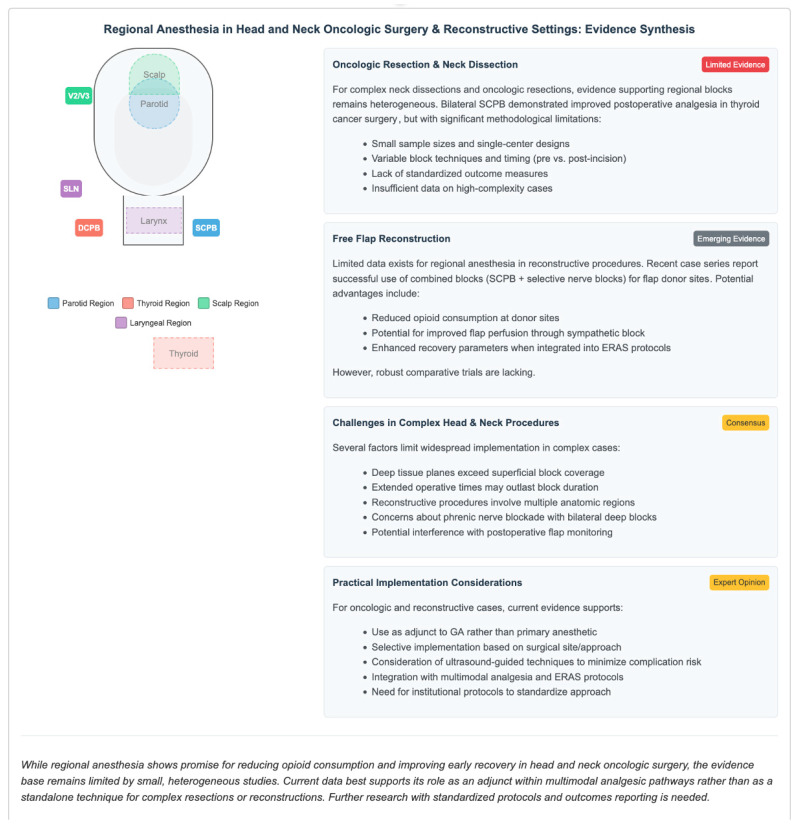
Regional anaesthesia in head and neck oncologic surgery and reconstructive settings: evidence synthesis [34,42].

Notably, this body of evidence is mostly procedural and pragmatic, as opposed to outcome-based, and unlike the thyroid literature, allows for less clear inference of postoperative analgesic efficacy. Importantly, this evidence is largely procedural and pragmatic rather than based on outcomes, which does not permit the same level of inference about postoperative analgesic efficacy as the thyroid literature (Figure 4).

This area is supported by stronger evidence than is sometimes assumed. A 2023 meta-analysis of randomised controlled trials noted that airway nerve blocks for awake tracheal intubation were associated with better intubating conditions, shorter intubation time, reduced cough and gag reflex, improved patient satisfaction and fewer overall complications compared with airway anaesthesia alone without nerve blocks [50]. These data compel us to take airway-related regional techniques out of the anecdotal realm and into what can be considered an evidence-supported application domain in head and neck anaesthesia, despite the fact that their outcome measures differ from those most often seen in studies on thyroidectomy.

#### 3.3.4. Oncologic Resections and Reconstructive Surgery

In more extensive procedures, including neck dissections, flap harvests, and major oncologic resections, the role of regional anaesthesia is less clearly defined [51,52]. With the available data in large oncologic resections and reconstructive surgery primarily derived from small observational cohorts or case series, or extrapolated from less complex procedures, regional techniques used in this context should at present be considered an adjunct to multimodal perioperative management rather than a stand-alone anaesthetic approach.

The role of regional anaesthesia in more extensive procedures such as neck dissections, composite oncologic resections, donor-site surgery and microvascular free-flap reconstruction is less clearly defined than thyroid surgery; however, the literature is growing. A retrospective cohort of head and neck microvascular reconstruction recipients where donor-site regional anaesthesia was associated with significantly shorter hospital stay, albeit opioid reduction did not reach significance [53]. Furthermore, a prospective randomised clinical trial in oral cavity free-flap reconstruction found that supplemental regional block anaesthesia resulted in significantly lower postoperative opioid use [54]. These results indicate that regional techniques may offer a clinically significant adjunct in select reconstructive and oncologic settings, particularly as part of opioid-sparing (or enhanced recovery) pathways. However, the evidence is less highly standardised compared to that of thyroidectomy, and expert consensus statements still highlight considerable variability in perioperative practice between institutions [55]. Currently, we do not have enough evidence to characterise the best block technique, dosing regimen or timing strategy for these intricate procedures.

### 3.4. Patient-Centred Outcomes and Analgesic Efficacy

This part is dedicated to patient-centred perioperative outcomes, such as postoperative pain, opioid consumption, quality of recovery, postoperative nausea and vomiting (PONV), stay time/hospital length of stay (LOS) and block-related safety.

To enhance interpretability, the results outlined below are organised by outcome domain and level of evidence with an emphasis on whether conclusions are based on randomised comparisons, observational analyses or descriptive case-based reports.

The deployment of regional anaesthesia in head and neck surgery has increasingly shifted attention from purely intraoperative use to longer-term analgesic outcomes and enhanced recovery metrics. Ultrasound-guided intermediate cervical plexus block (ICPB) was associated with superior 24 h analgesia compared to superficial SCPB in the head and neck region, with similar rates of complications [47].

#### 3.4.1. Postoperative Pain Scores

The reduction in postoperative pain is one of the most common areas in which regional anaesthesia has been reported to be favourable in head and neck surgery, although the robustness and consistency of this finding vary greatly between procedures performed and study design. Specifically, in thyroid surgery, multiple randomised controlled trials describe decreased early postoperative pain scores achieved with bilateral SCPB compared to general anaesthesia alone, and these results are consistent with meta-analytic data. Yet this apparent advantage is confined mostly to the first postoperative 24 h, and the clinical significance of this effect is study-dependent based on the type of pain scale used, baseline systemic analgesia, block technique and use of adjuvants. Outside of thyroid surgery, pain-related conclusions are based more on smaller observational or procedure-specific studies than on other direct evidence that is complementary but less definitive. Thus, pain reduction seems most plausible for SCPB in thyroid and select superficial cervical operations, while extrapolation to more extensive head and neck surgery should be tempered. The greatest effect is consistently seen in the first 24 h after surgery, with only marginal benefit thereafter. A recent prospective cohort study found that bilateral SCPB improved postoperative analgesia in thyroid cancer surgery, which had a more complex dissection [52]. An RCT with 100 thyroidectomy patients showed that bilateral SCPB and general anaesthesia (GA) resulted in lower pain scores and usage of morphine compared to GA alone [39]. A similar randomised trial of carotid endarterectomy also demonstrated that SCPB reduced postoperative morphine consumption by 50% and improved patient satisfaction [56]. A more recent study similarly randomised 140 patients undergoing radical thyroid cancer surgery to GA alone vs. GA + bilateral SCPB with adjunct adjuvants (dexmedetomidine or dexamethasone); the adjunct groups had lower intra- and postoperative analgesic use, better resting and active VAS scores, and improved recovery metrics [57]. In another double-blind trial of 100 patients, bilateral SCPB significantly reduced pain scores in the first 24 h post-thyroidectomy [42]. The greatest effect is consistently seen in the first 24 h after surgery, with only marginal benefit thereafter.

#### 3.4.2. Opioid-Sparing Effects and Multimodal Analgesia

Minimising opioid use is a primary objective of enhanced recovery after surgery (ERAS) pathways. In the literature, there is evidence that regional blocks for head and neck surgery can be an important component of opioid-sparing. Some authors have described the use of either SCPB or ICPB in conjunction with general anaesthesia for the purpose of reducing postoperative pain and opioid consumption during wide-field surgeries. A single randomised trial in modified radical mastoidectomy showed decreased opioid and analgesic medication usage with ultrasound-guided SCPB compared to no block [58]. In a more recent RCT, when anterior cervical spine surgery was performed and ICPB vs. cervical erector spinae block compared, the authors noted that though onset of block had occurred in less time within the ICPB group during recovery, the nalbuphine consumption by this group was also higher, revealing the complex implications of block type on opioid utilisation [31]. In a randomised controlled trial of SCPB utilising 0.25% bupivacaine in thyroidectomy, its use was associated with reduced intraoperative fentanyl and postoperative morphine requirements compared to placebo [41]. An additional study from China, opioid-free anaesthesia with ultrasound-guided ICPB offered better recovery and lower nausea and pain scores compared to an opioid-based anaesthesia [59]. These results underscore the importance of regional blocks being placed within a multimodal protocol for analgesia and not considered as a standalone approach.

#### 3.4.3. Patient-Reported Recovery and Functional Outcomes

Patient-reported outcomes, such as quality of recovery or functional restoration, are increasingly evaluated beyond analgesia. Studies utilising superior laryngeal nerve block or recurrent laryngeal nerve block as adjuvant techniques in awake fibre-optic intubation or minimally invasive laryngeal interventions demonstrate enhanced patient tolerance and decreased sedative requirements [33]. In a randomised study of patients undergoing anterior cervical discectomy or fusion, the addition of SCPB to GA improved QoR-40 scores at 24 h compared to GA alone [60]. In another report of occipital and mastoid surgery, SCPB reduced pain associated with movement and allowed for earlier ambulation and comfort during aural care [61]. Despite limited and less well-studied long-term outcomes (i.e., chronic pain or functional impairment), there is early evidence to support faster recovery profiles with regional techniques potentially improving patient experience and hospital stay.

#### 3.4.4. Impact on Length of Stay, PONV, and Other Metrics

A systematic review and meta-analysis recently confirmed that SCPB can reduce early postoperative pain and decrease hospital stay after thyroid surgery [44]. Some studies report secondary benefits, including shorter hospital stay, lower postoperative nausea and vomiting (PONV), and earlier readiness for discharge, but these findings are less consistently reported than pain outcomes and are particularly vulnerable to confounding from differences in ERAS pathways, discharge criteria, anaesthetic technique, and institutional practice. For example, in a radical thyroid cancer surgery study the SCPB + adjuvants had less time to first analgesic request, less PONV and improved discharge metrics [57]. A retrospective study in paediatric otologic surgery [62], although powered for pain outcomes, revealed that SCPB reduced the incidence of PONV and analgesic use. Nevertheless, the size of these effects across the board is usually small and typically only apparent in the early postoperative period; further variation in surgical procedures and analgesic regimens makes definitive conclusions difficult.

#### 3.4.5. Safety Profile and Block-Related Complications

From a safety viewpoint, most studies report no serious block-related complication when ultrasound guidance is utilised. A rigorous trial mixing superficial and deep blocks in high-risk subjects submitted to parathyroidectomy also found no impairment of respiratory function [45]. A strong safety profile for regional head and neck blocks has been described when these blocks are performed under ultrasound guidance by experienced operators. In a systematic review of SCPB techniques no major block-related complications occurred in large series but these small risks still exist (e.g., local anaesthetic systemic toxicity, phrenic nerve block and haematoma formation in anticoagulated patients) [22]. Among high-risk patients undergoing parathyroidectomy, a protocol of ultrasound-guided bilateral superficial and deep cervical plexus blocks in addition to general anaesthesia provided improved analgesia without impairment of respiratory function [45]. Notably, there were no respiratory compromise or significant morbidity due to the block in trials of SCPB in thyroid and cervical spine surgery [44,60].

Superficial approaches tend to have a low major complication burden with careful technique compared to deep and combined blocks, but the latter hold greater concern for phrenic nerve palsy, intravascular injection, epidural or subarachnoid spread, and unexpected recurrent laryngeal or other cranial nerve effects [61,62]. Comparison with a series of carotid surgeries indicates that deep/combined techniques carry a more serious risk of needle-related complications than superficial/intermediate approaches [42]. Furthermore, randomised imaging-based data show that the depth of an injection correlates with the degree of diaphragmatic dysfunction, and techniques at depth produce greater impairment of ipsilateral diaphragmatic motion [43,44,63]. And significantly, the intermediate cervical plexus block may occupy a position in between: some studies show no hemidiaphragmatic paresis when low-dose ultrasound-guided protocols are used, whereas others have shown clinically relevant and measurable diaphragmatic dysfunction depending on what concentration or injection strategy is used. But the evidence for deeper blocks (e.g., deep cervical plexus) remains weaker, and complication rates appear to increase with combined techniques or bilateral techniques in high-risk patients.

#### 3.4.6. Limitations of Current Evidence

While results are encouraging, several limitations should limit interpretation. A meta-analysis found that bilateral SCPB had a modest benefit in terms of decreased postoperative analgesic consumption and better early pain scores, but the study heterogeneity was significant [43]. Most studies are small, single-centre, heterogeneous surgical populations (thyroid, cervical spine, mastoid, and carotid), and none include long-term follow-up. However, the data are heterogeneous with differing block techniques, local anaesthetic volume, timing and endpoints. The main limitations are small sample size, single-centre design and no long-term outcomes. Further, differences in block technique (ultrasound vs. landmark), volumes of local anaesthetic used, adjuvant use and definitions of outcome all contribute to the difficulty in performing meta-analysis. Other trials (e.g., ICPB vs. erector spinae block) showed no difference or demonstrated a paradoxical increase in opioid use, suggesting that choice of block matters [31]. Timing was also variable; the studies evaluate pre-incisional pre-emption, post-induction blocks or postoperative rescue strategies. In addition, endpoints lack standardisation. Pain scores are evaluated at varying timepoints, opioid consumption is expressed using heterogeneous comparators or conversion metrics and functional recovery outcomes are unnaturally recorded. These factors limit the ability to do direct comparison of studies, and likely explain why positive findings that appear robust under one set of circumstances are not often reproduced under different numerical regimes or environmental conditions.

This issue is particularly salient for safety interpretation, since the risk of complications is strongly confounded by whether studies include superficial, intermediate, deep, or combined techniques; unilateral vs. bilateral performance; and low-volume vs. higher-concentration injectate strategies [44,63]. Consequently, the term ‘cervical plexus block’ usually hides clinically significant differences in risk.

Large oncologic resections and flap harvests and ambulatory head and neck surgery settings, on the other hand, continue to be relatively under-explored.

#### 3.4.7. Practical Implications and Recommendations for Clinicians

A pathway-oriented rather than technique-only integrated model of regional anaesthesia into the practice of head and neck surgery should be pursued. Preoperatively, clinicians should clarify the intended primary effect of the block, assess airway/respiratory reserve and history of previous neck surgery or prior neck radiotherapy; consider infective status/anticoagulant therapy/history; and determine if the anticipated source of pain is superficial/focal/deep. The least invasive technique that provides coverage of the operative field should generally be preferred intraoperatively, and the block should be included in standardised multimodal analgesia rather than utilised as a substitute for it. Additional specific, quantifiable endpoints such as opioid consumption, nausea/vomiting, airway stability and readiness to mobilise should be used within the context of utilising pain scores alone when considering postoperative success in achieving enhanced recovery goals. Most importantly, regional anaesthesia should be incorporated into ERAS-type perioperative care pathways vs. simply a case of an isolated intervention in high-complexity surgery (particularly major oncologic or reconstructive pathways).

### 3.5. Training, Learning Curve, and Implementation

The safe and effective application of regional anaesthesia in head and neck surgery relies not only on anatomical and technical knowledge but equally on structured training and competence acquisition.

#### 3.5.1. Learning Curves and Competency Acquisition

**Figure 5:**
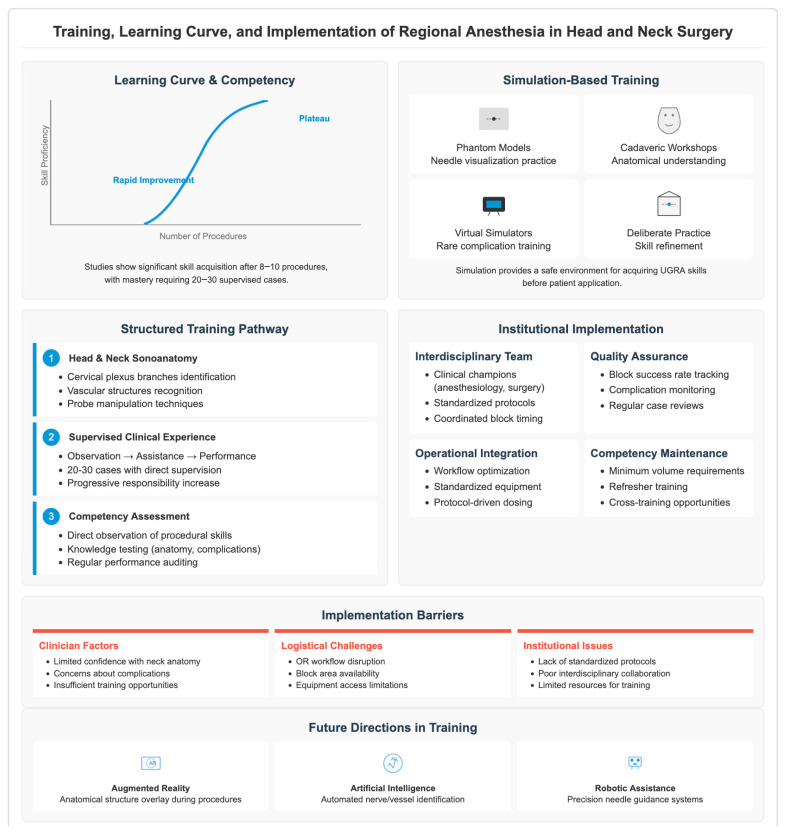
Training, learning curve, and implementation of regional anaesthesia in head and neck surgery [51,52,53,54].

UGRA involves developing shared psychomotor and visuospatial skills (e.g., probe orientation, needle-tip tracking, and sonoanatomy interpretation). Initial studies of simulation models for US-guided procedures showed that novices improved similar metrics in speed and accuracy even over a few trials. In a phantom study of ultrasound-guided simulated nerve blocks, for example, naive participants plateaued in accuracy and time performance after ~8 trials [63]. More generally, the literature describes competency acquisition in UGRA as following a sigmoidal learning curve: an early period of rapid improvement followed by a slower trajectory (plateau) phase [64] (Figure 5).

This realisation highlights the importance of structured practice, feedback and reflective work over opportunistic “on-the-job” learning.

#### 3.5.2. Simulation and Structured Courses

Simulation-based training provides a safe, reproducible environment to learn key UGRA skills prior to patient care. A one-day simulation course designed specifically for emergency physicians showed statistically significant improvements in self-rated knowledge in technique indication, equipment and local anaesthetic toxicity management domains [65]. Although those courses generally cover UGRA as a whole, that basic theory is extrapolative to head and neck regional approaches (e.g., cervical plexus blocks and selective nerve blocks). Deliberate practice, particularly of critical skills (device familiarisation, image optimisation, needle advancement), has been advocated using phantoms, cadaveric models and ultrasound simulators [66]. Importantly, simulation facilitates exposure to rare events/complications (e.g., intravascular injection and needle misdirection) in a controlled environment.

#### 3.5.3. Interdisciplinary and Institutional Implementation

Head and neck regional anaesthesia requires a specialised training curriculum focused on addressing this unique anatomy, airway, and surgical characteristics [67]. These programmes should encompass a thorough introduction to head and neck sonoanatomy: the cervical plexus, superficial and deep branches, and pertinent cranial nerve adjuncts; along with practical modules for manipulation of the ultrasound probe taking into account bony, vascular and airway structures [23]. In tandem, accurate needle path planning to avoid injury to key structures (e.g., the carotid artery, vertebral vessels, or the phrenic nerve) is as crucial as rehearsed intraoperative workflows (regarding sedation strategies while balancing airway rescue capabilities and right timing of blocks for surgery) [25]. Some authors highlight both the complexity of head and neck anatomy and their proximity to the treatment airway (mentioned further above), which requires more vigilance and multidisciplinary teamwork on behalf of the anaesthesiologists and the head and neck surgeons than most peripheral nerve blocks. For successful institutional adoption of regional anaesthesia in head and neck surgery, collaboration among departments (anaesthesia, otolaryngology, nursing, pain management) is also key. Identifying champion clinicians and developing a “head and neck regional anaesthesia” pathway should spearhead this process, followed by establishing standardised protocols for block execution, patient monitoring, and documentation [68]. Perioperative protocols, with formal training and simultaneous participation of anaesthetic and surgical teams, are warranted to optimise the pre-defined expectations on timing, analgesic adjuvants and intraoperative management. Monitoring block success rates, complication incidence, and analgesic outcomes as ongoing audits provides the feedback necessary to improve practice and develop institutional expertise. Well-structured approaches involving formal training paired with proctoring and performance feedback show greater adoption and safety outcomes than informal, ad hoc approaches.

#### 3.5.4. Credentialing, Quality Assurance and Maintenance of Competency

From a quality assurance viewpoint, credentials of providers delivering head and neck regional blocks should follow the same frameworks as other procedures: published supervised cases (e.g., 20–30 supervised cases), evaluation of complication recognition skills, routine performance audit and refresher simulation sessions. UGRA education recommends maintaining the highest standard of competence through continued practice, complication review and continuing education [69]. Due to the relative infrequency of some head and neck block techniques vs. common limb blocks, maintaining volume and proficiency may be somewhat difficult. Institutions should monitor block volumes, promote cross-training and conduct periodic simulations to reinforce skills retention [70].

#### 3.5.5. Barriers to Implementation and Future Directions in Training

Various challenges to the widespread implementation of head and neck regional anaesthesia are reported. Barriers noted to implementation commonly involved: operator inexperience and lack of confidence regarding neck anatomy under US (ultrasound); reluctance by surgical teams not used to such regional pathways; logistical issues including scheduling a block before incision or making the block work within theatre workflow; and limited institutional willingness to support training and equipment. A review of education in ultrasound-guided nerve blocks highlighted that despite growing interest in this area, many training programmes remain fragmented and lack standardised curricula and assessment tools [25]. Overcoming these barriers demands institutional will, reserved training time and inter-professional culture shift. In the future, newly emerging technologies promise to improve training in head and neck regional anaesthesia. The future may hold augmented reality (AR) overlays for needle guidance, virtual-reality (VR) simulators and artificial intelligence (AI)-driven ultrasound image segmentation to potentially improve training efficiency and standardise assessment [31]. AR systems have been used, for example, to follow instructions for ultrasound-guided regional techniques with remote direct observations [25]. Moreover, competency-based training models, digital logbooks and performance analytics are set to allow more individualised education and assessment pathways tailored to head and neck regional anaesthesia.

## 4. Discussion

### 4.1. Emerging Landscape

The emerging landscape of regional anaesthesia for head and neck surgery still has some important limitations and controversy, while several growth opportunities exist.

First, the existing body of literature is skewed towards observational studies, small randomised trials, heterogeneous patient populations and variable outcome measures. An example of recent studies highlighting the importance and complexity of these techniques includes a randomised trial comparing ultrasound-guided intermediate cervical plexus block vs. cervical erector spinae block in anterior cervical spine surgery that found no superiority of one approach [31]. The best data come from thyroid surgery, for which randomised trials and meta-analyses generally show a borderline early analgesic benefit of SCPB and some decrease in postoperative opioid use. In contrast, the literature for salivary gland, airway-related, oncologic, and reconstructive procedures was heavily weighted toward observational studies, small cohorts, and case-based reports that are especially susceptible to selection bias and confounding. There are significant differences in block protocols (volume given, concentration used, timing of block relative to surgery), outcome definitions (e.g., morphine equivalents, pain scores, and functional recovery) and long-term follow-up (e.g., chronic pain), meaning the results are difficult to synthesise across studies. This underlines the poor generalisability of the present results. Second, given the complexity of head and neck surgeries involving intricate anatomical structures, airway obstruction and large tissue reconstructions, regional block experience in this setting remains limited. In a systematic review on regional anaesthesia in head and neck surgery, there was predominance of superficial procedures with very little representation for flap or oncologic surgery [25].

Evidence now evolves to parotid, awake airway and microvascular oncologic reconstruction. Importantly, though, these domains are methodologically heterogeneous and often target different clinical endpoints. For instance, studies involving airway nerve block primarily focus on intubation conditions and patient tolerance, whereas free-flap reconstruction studies are more concerned with opioid exposure, donor-site pain and duration of hospital stay. This, in part, helps explain why this literature seems less cohesive than the evidence on thyroidectomy and why there needs to be procedure-specific interpretation [48,54].

This limits applicability in the high-complexity surgical realm.

First, the evidence base is heterogeneous, not just partitioned by classes but hierarchically. In fact, even in seemingly similar studies, there are significant differences in block depths, ultrasound guidance, laterality, local anaesthetic regimens or administration of other adjuvants and their timing relative to incision (or induction). As a result, pooled interpretation across the larger head and neck field remains challenging and generalisability to complex surgery is still low.

### 4.2. Controversies in Technique and Safety

A major contention revolves around the exchange of effective block depth, anaesthetic coverage and inadvertent dissemination. Although deeper cervical plexus techniques may theoretically provide more extensive sensory and motor coverage, the current evidence suggests that this is at the cost of safety. Systematic review shows that deep or combined blocks are significantly associated with a higher rate of serious needle-related complications as compared to superficial or intermediate approaches in carotid surgery [43]. Similarly, randomised physiologic data demonstrate that phrenic nerve dysfunction is depth-dependent, with deep block resulting in the greatest impairment of ipsilateral diaphragmatic motion [43,44]. This has particular implications for anaesthetists planning bilateral blocks or managing patients with poor respiratory reserve. Alternatively, intermediate blocks remain an area of contention, with some ultrasound-guided low-dose paradigms seemingly circumventing clinically relevant hemidiaphragmatic paresis, whereas others demonstrate quantifiable diaphragm dysfunction in response to injectate concentration and spread. Thus, the current controversy is not merely about whether one technique is ‘safe’ and another is ‘unsafe’, but rather how to maximise coverage whilst minimising spread to the phrenic nerve, neuraxis and adjacent vascular or airway structures [71]. Debate remains whether intermediate (compared with deep) cervical plexus blocks do in fact spare the phrenic nerve or bypass epidural/vertebral spread [23]. There is still limited safety data, particularly for bilateral or deep approaches in head and neck surgery. Although regional anaesthesia overall can be considered safe, concern remains: a recent review states that only about one-fifth of consultant anaesthetists in the UK feel confident performing both sides of all blocks, citing fear of complications as a barrier [72]. There is a lack of standardised complication reporting, making it difficult to accurately estimate rare but serious events such as phrenic palsy, airway compromise and local anaesthetic systemic toxicity. In head and neck surgery, the close proximity of major vessels and airway structures to cranial nerve anatomy adds to these concerns.

### 4.3. Variability in Clinical Application and Workflow

Challenges also include the integration of regional anaesthesia into head and neck surgical pathways. There is a wide variability in the practice of block timing (pre-incision vs. post-induction), sedation strategies, coordination with surgical workflow and postoperative analgesic protocols across institutions and studies. Some authors mention that operational factors (availability of block-room, training and equipment), more than pure clinical evidence, limit adoption [11]. Established blocks (e.g., superficial cervical plexus block) also may not reach their maximum benefit without institutional pathways and team alignment [73,74,75,76].

### 4.4. Practical Implications for Clinicians

An interpretation of the current literature that is clinically useful is to match each regional technique with its predominant perioperative intent. If early postoperative analgesia is the goal in a superficial cervical procedure, approaches initiated at the level of the superficial cervical plexus currently have the greatest backing. In the case of awake airway management, selective airway nerve blocks are preferable to cervical plexus techniques. If minimisation of opioid use is the primary target and goal in major oncologic reconstruction, there is a sound body of evidence that suggests regional anaesthesia should be reserved for inclusion as one component in multimodal and ERAS-based perioperative care rather than implemented as a stand-alone intervention with deterministic novelty. Surgical learning is more intricate and nuanced, and this procedural framework minimises overgeneralisation and better translates anatomy into practice. Evidence continues to develop, and clinicians need to take a balanced, pragmatic stance: choose appropriate patients (expected profound benefit, minimal respiratory compromise), use ultrasound-guided approaches with standardised protocols of practice for blocks, capture outcomes and complications followed by collaboration with head and neck surgeons on block timing/preemptive or postoperative analgesic strategies and audit/research network participation which will allow contribution to evidence generation. At present, the clearest clinical message is that regional anaesthesia should be matched to procedure complexity and evidence strength. It is best supported as an adjunct in selected superficial cervical procedures, while its use in deeper, bilateral, or reconstructive settings should be individualised until more standardised comparative data become available.

## 5. Conclusions

When utilised in the perioperative setting, regional anaesthesia proves to be an effective adjunct to head and neck surgery, providing excellent analgesia during the procedure while minimising opioid consumption and improving discharge profiles. Its success relies on a clear understanding of anatomy, an adequate ultrasound and collaboration between anaesthesiologist and surgeon. Although positive evidence currently exists for superficial neck approaches, more widespread applications in complex oncologic and reconstructive paradigms are lacking. The future advancement of processes will be highly dependent upon standardised methodologies with rigorous clinical trials, coupled with refined teaching supported via simulation and technological advances.

The currently strongest evidence supports superficial cervical plexus-based approaches for use in thyroid and parathyroid surgery; however, growing data within parotid surgery, airway-related interventions and microvascular reconstructive oncology suggest that regional anaesthesia can also play a wider galley role as an adjunct beyond the thyroid field if appropriate, given the operative site and perioperative aim.

Regional anaesthesia, in the end, is a critical milestone towards accuracy, safety and patient-focused management in state-of-the-art head and neck surgery.

## Figures and Tables

**Table 1:** Techniques, advantages and potential limitations.

Technique	Target Nerves/Plexus	Main Surgical Applications	Advantages	Potential Risks/Limitations
Superficial Cervical Plexus Block (SCPB)	Superficial branches of C2–C4 (great auricular, lesser occipital, transverse cervical, supraclavicular)	Thyroidectomy, parotidectomy, neck dissection, carotid endarterectomy	Easy to perform, minimal motor blockade, reduced opioid use	Limited coverage for deep tissues, possible local haematoma
Intermediate Cervical Plexus Block (ICPB)	Intermediate plane between investing and prevertebral fascia, targeting C2–C4 roots	Carotid surgery, deep neck dissections, reconstructive flaps	Broader field of analgesia than SCPB	Risk of phrenic nerve block, inadvertent deep spread
Deep Cervical Plexus Block (DCPB)	C2–C4 deep branches near transverse processes	Major neck dissection, cervical spine surgery	Strong, long-lasting analgesia	Higher complication rate (phrenic, vertebral artery, epidural spread)
Superior Laryngeal Nerve Block (SLNB)	Internal branch of superior laryngeal nerve (from vagus)	Awake intubation, supraglottic surgery	Preserves airway reflexes, improves comfort	Hoarseness, aspiration risk if bilateral
Recurrent Laryngeal Nerve Block (RLNB)	Terminal branch of vagus nerve	Tracheal surgery, subglottic procedures	Facilitates airway manipulation	Bilateral block risks airway obstruction
Greater Occipital Nerve Block (GONB)	Dorsal ramus of C2	Posterior scalp incisions, occipital pain	Simple, low complication rate	May not cover anterior regions
Sphenopalatine Ganglion Block (SPGB)	Parasympathetic fibres via maxillary nerve (V2)	Endoscopic sinus surgery, rhinoplasty	Reduces intraoperative bleeding, analgesia	Short duration, limited to nasal territory
Hybrid/Combined Techniques	Multi-nerve targeting (e.g., SCPB + SLNB)	Extended neck dissection, flap harvest	Synergistic analgesia, less general anaesthesia needed	Technical complexity, cumulative risk of LA toxicity

## Data Availability

No new data were created or analysed in this study. Data sharing is not applicable to this article.
